# Ultrarestrictive Opioid Prescription Protocol for Pain Management After Gynecologic and Abdominal Surgery

**DOI:** 10.1001/jamanetworkopen.2018.5452

**Published:** 2018-12-07

**Authors:** Jaron Mark, Deanna M. Argentieri, Camille A. Gutierrez, Kayla Morrell, Kevin Eng, Alan D. Hutson, Paul Mayor, J. Brian Szender, Kristen Starbuck, Sarah Lynam, Bonnie Blum, Stacey Akers, Shashikant Lele, Gyorgy Paragh, Kunle Odunsi, Oscar de Leon-Casasola, Peter J. Frederick, Emese Zsiros

**Affiliations:** 1 Department of Gynecologic Oncology, Roswell Park Comprehensive Cancer Center, Buffalo, New York; 2 Department of Pharmacy, Roswell Park Comprehensive Cancer Center, Buffalo, New York; 3 University at Buffalo Jacobs School of Medicine and Biomedical Sciences, Buffalo, New York; 4 Department of Biostatistics and Bioinformatics, Roswell Park Comprehensive Cancer Center, Buffalo, New York; 5 Department of Dermatology, Roswell Park Comprehensive Cancer Center, Buffalo, New York; 6 Division of Pain Medicine, Roswell Park Comprehensive Cancer Center, Buffalo, New York

## Abstract

**Importance:**

Opioids are routinely prescribed for postoperative home pain management for most patients in the United States, with limited evidence of the amount needed to be dispensed. Opioid-based treatment often adversely affects recovery. Prescribed opioids increase the risk of chronic opioid use, abuse, and diversion and contribute to the current opioid epidemic.

**Objective:**

To evaluate whether after hospital discharge, postsurgical acute pain can be effectively managed with a markedly reduced number of opioid doses.

**Design, Setting, and Participants:**

In this case-control cohort study, an ultrarestrictive opioid prescription protocol (UROPP) was designed and implemented from June 26, 2017, through June 30, 2018, at a single tertiary-care comprehensive cancer center. All patients undergoing gynecologic oncology surgery were included. Patients undergoing ambulatory or minimally invasive surgery (laparoscopic or robotic approach) were not prescribed opioids at discharge unless they required more than 5 doses of oral or intravenous opioids while in the hospital. Patients who underwent a laparotomy were provided a 3-day opioid pain medication supply at discharge.

**Main Outcomes and Measures:**

Total number of opioid pain medications prescribed in the 60-day perioperative period, requests for opioid prescription refills, and postoperative pain scores and complications were evaluated. Factors associated with increased postoperative pain, preoperative and postoperative pain scores, inpatient status, prior opioid use, and all opioid prescriptions within the 60-day perioperative window were monitored among the case patients and compared with those from consecutive control patients treated at the center in the 12 months before the UROPP was implemented.

**Results:**

Patient demographics and procedure characteristics were not statistically different between the 2 cohorts of women (605 cases: mean [SD] age, 56.3 [14.5] years; 626 controls: mean [SD] age, 55.5 [13.9] years). The mean (SD) number of opioid tablets given at discharge after a laparotomy was 43.6 (17.0) before implementation of the UROPP and 12.1 (8.9) after implementation (*P* < .001). For patients who underwent laparoscopic or robotic surgery, the mean (SD) number of opioid tablets given at discharge was 38.4 (17.4) before implementation of the UROPP and 1.3 (3.7) after implementation (*P* < .001). After ambulatory surgery, the mean (SD) number of opioid tablets given at discharge was 13.9 (16.6) before implementation of the UROPP and 0.2 (2.1) after implementation (*P* < .001). The mean (SD) perioperative oral morphine equivalent dose was reduced to 64.3 (207.2) mg from 339.4 (674.4) mg the year prior for all opioid-naive patients (*P* < .001). The significant reduction in the number of dispensed opioids was not associated with an increase the number of refill requests (104 patients [16.6%] in the pre-UROPP group vs 100 patients [16.5%] in the post-UROPP group; *P* = .99), the mean (SD) postoperative visit pain scores (1.1 [2.2] for the post-UROPP group vs 1.4 [2.3] for pre-UROPP group; *P* = .06), or the number of complications (29 cases [4.8%] in the post-UROPP group vs 42 cases [6.7%] in the pre-UROPP group; *P* = .15).

**Conclusions and Relevance:**

Implementation of a UROPP was associated with a significant decrease in the overall amount of opioids prescribed to patients after gynecologic and abdominal surgery at the time of discharge for all patients, and for the entire perioperative time for opioid-naive patients without changes in pain scores, complications, or medication refill requests.

## Key Points

**Question:**

Can postoperative pain be appropriately managed after hospital discharge with minimal or no opioids in patients undergoing gynecologic and abdominal surgery?

**Findings:**

In this case-control cohort study of 1231 patients undergoing gynecologic oncology surgery, implementation of an ultrarestrictive opioid prescription protocol was associated with a significant decrease in the number of opioids dispensed at discharge. Using the ultrarestrictive opioid prescription protocol to manage postoperative pain was also associated with a significant decrease in the number of opioids dispensed during the entire perioperative period for opioid-naive patients without changes in postoperative pain scores, complications, or increases in the number of refill requests.

**Meaning:**

A reduction in the number of opioids dispensed for postoperative pain management is safe and attainable.

## Teaser

This case-control cohort study compares the total number of opioid pain medications prescribed after gynecologic and abdominal surgery, the number of requests for opioid prescription refills, and postoperative pain scores and complications before and after implementation of an ultrarestrictive opioid prescription protocol.

## Introduction

The most recent report from the Centers for Disease Control and Prevention estimates that 116 people in the United States die per day after an opioid overdose and that 40% of all deaths due to an opioid overdose involve a prescription opioid.^1^ The number of deaths due to a prescription opioid overdose totaled an estimated 20 000 in 2015, and more than 4.5 million people in the United States misuse prescription opioids.^2^ An executive report from the US Council of Economic Advisers estimated that the total economic burden of the opioid crisis in 2015 was $504.0 billion, or 2.8% of the gross domestic product.^3^ Given these statistics, the US federal government declared the opioid epidemic a public health emergency. Thus, all health care professionals are encouraged to take appropriate steps to decrease the number of opioids dispensed and monitor their misuse, abuse, and diversion.

An estimated 48 million people in the United States undergo surgical procedures annually, and opioids are still the primary modality for managing acute postoperative pain.^4^ Large numbers of opioid pills, mainly oxycodone hydrochloride and hydrocodone bitartrate, are often prescribed to patients after surgery, with limited evidence of the amount necessary for adequate pain management and optimal recovery.^5^ Studies suggest that patients take only 28% of the prescribed doses of opioids.^6^ New persistent opioid use is a common and underappreciated surgical complication, which occurs among 5.9% of patients undergoing minor surgery and in 6.5% of those having major surgery.^7^ These findings indicate that postsurgical opioid use could also be a gateway to chronic opioid use and provide a strong justification to limit perioperative opioid use.

Within the last decade in the United States, many surgical subspecialties have adopted enhanced recovery after surgery (ERAS) protocols to improve patient recovery. Enhanced recovery after surgery protocols focus on multimodal analgesia, with medications administered preoperatively, intraoperatively, and immediately after surgery that target different pain pathways to limit opioid use during hospital care.^8^ These efforts attempt to minimize the adverse effects of opioids (sedation, nausea, vomiting, ileus, respiratory depression, and pruritus), which can negatively affect the recovery of a patient. Unfortunately, ERAS protocols often omit postdischarge patient care; thus, many surgical patients are still prescribed an excessive amount of opioid pain medications for home use.^6^

Prior to June 2017, the Department of Gynecologic Oncology at our large tertiary care center in Western New York (Roswell Park Comprehensive Cancer Center) routinely and liberally prescribed opioids to most patients to manage their postoperative pain, similar to other gynecologic oncology practices in the country. Our department had previously adopted ERAS recommendations to maximize nonopioid medications while patients were in the hospital, but we lacked guidelines on pain management after hospital discharge. We hypothesized that most of our surgical patients were prescribed opioid pain medication in excessive amounts despite following our state regulation for not providing more than a 7-day supply of opioids for acute pain at discharge for patients with no diagnosis of cancer.^9^ We also hypothesized that the postoperative pain in our patients who underwent ambulatory and minimally invasive surgery could be adequately managed with nonopioid pain medication without negatively affecting recovery. Thus, in June 2017, our department designed and implemented an ultrarestrictive opioid prescription protocol (UROPP) for all patients undergoing minor or major procedures, regardless of their diagnosis and whether they were opioid naive or chronic opioid users. Our goal was to test whether such a radical approach is safe, is sustainable, and could lead to a marked decrease in the number of opioids dispensed during the postoperative period.

## Methods

### The UROPP

**Figure 1.**
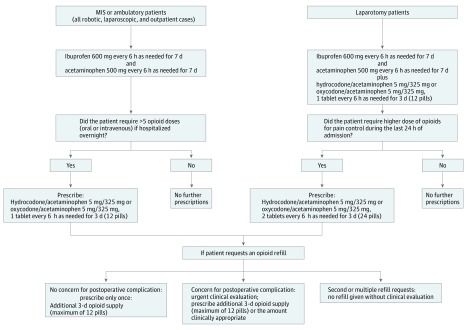
Ultrarestrictive Opioid Prescription Protocol The algorithm for prescribing postoperative pain medications at the time of discharge and handling refill requests is shown on the left for minimally invasive surgical (MIS) and ambulatory cases and on the right for laparotomy cases. The protocol remained the same regardless of prior opioid use.

The UROPP was developed based on patient feedback in conjunction with recommendations from surgeons and pain specialists and was implemented in June 26, 2017, through June 30, 2018, for all surgical patients at our department. Based on the comments from our postoperative patients, who claimed to use very little of the prescribed opioids and were frequently inquiring about returning the unused pills, we decided to test whether opioids were even necessary after a minor or minimally invasive procedure. We also observed that patients who underwent a laparotomy used less opioids by the day of discharge (typically 3-4 days after surgery); thus, we thought that adding an additional 3-day supply after discharge would be sufficient. Rather than relying on patients returning the unused medications to count the leftover pills, as a multidisciplinary team, we agreed to test a more radical approach and assess how much additional medication was needed after limited initial discharge medications. The steps of the UROPP are outlined in Figure 1. The Roswell Park Comprehensive Cancer Center Institutional Review Board provided approval for clinical data collection, but because the change in opioid prescribing practice was considered a quality improvement study, it was deemed exempt from approval and patient consent as no intervention was performed. This study followed the Strengthening the Reporting of Observational Studies in Epidemiology (STROBE) reporting guidelines.

In brief, all surgical patients underwent a short preoperative counseling session conducted by the surgeon or nursing staff about management of postoperative pain, which included discussion of the expected discharge pain medications (per protocol), the adverse effects of opioid pain medications, and alternative methods of pain control. All opioid pain medications were counted for the duration of admission. Per protocol, all patients undergoing ambulatory surgery or minimally invasive surgery (MIS; all laparoscopic and robotic cases regardless of comprehensive cancer staging) were prescribed a nonopioid pain relief regimen consisting of a 7-day supply of ibuprofen and acetaminophen as needed at hospital discharge. If, while hospitalized, the patient required more than 5 opioid doses (oral or intravenous) in the 24 hours prior to discharge, then the patient was prescribed a 3-day supply of an opioid pain medication as needed, which totaled 12 tablets (hydrocodone and acetaminophen or oxycodone and acetaminophen).

Patients who underwent a laparotomy were equally prescribed ibuprofen and acetaminophen with the addition of a 3-day supply of an oral opioid as needed (hydrocodone and acetaminophen or oxycodone and acetaminophen), for a total of 12 tablets. If patients in the laparotomy group displayed an increased need for opioids in the 24 hours prior to discharge (>5 opioid doses given orally or intravenously), then the patient was discharged home with an additional 3-day supply of an opioid pain medication, for a maximum of 24 tablets. All telephone calls for refill requests were tracked. To ensure that the postsurgical pain was well controlled, we clarified with patients that they can request opioid refills if needed. If a refill was requested over the telephone, then the patient was triaged for an urgent postoperative visit if the pain was thought to be potentially related to evolving postsurgical complications. Only an additional 3-day supply (12 pills) was provided for refill. There were no second refills provided without a clinical evaluation (Figure 1). Patients with a contraindication to ibuprofen or acetaminophen were given only the drug to which they did not have a contraindication, or they were provided with a maximum 3-day supply of opioids, if it was thought to be clinically indicated.

Adherence to the protocol was tracked in real time by inpatient pharmacists who monitored all outgoing prescriptions for compliance. All deviations from protocol were immediately captured, and the physicians were asked to document indications for deviation. A total of 22 of 627 original post-UROPP cases were removed from analysis owing to protocol deviations because the effect of limited prescribed opioids at discharge could not be evaluated in these patients. These 22 patients (3.5% of the total) were discharged home with a higher number of opioids owing to a physician’s noncompliance with the protocol (eg, violations occurred owing to a new trainee forgetting about the protocol, patients who underwent ambulatory surgery with large excisions, or chronic opioid users requesting opioids after an ambulatory surgery). Most (16 of 22 [72.7%]) of these deviations occurred within the first 3 months of protocol implementation. Removing these 22 patients from the analysis did not significantly change any of our findings, and the total opioid-sparing effect during the 12 months of the study with these 22 patients also included in the analysis is shown in the eTable and eFigure in the Supplement.

### Patient Cohort

This study was designed as a retrospective control and prospective case cohort design with 626 controls (pre-UROPP, July 1, 2016, to June 25, 2017) and 605 cases (post-UROPP, June 26, 2017, to June 30, 2018) of all gynecological oncology patients (all women) undergoing major or minor surgery at a tertiary-care National Cancer Institute–designated comprehensive cancer center. Surgical cases ranged from minor outpatient resections and hysteroscopy cases to comprehensive robotic or laparoscopic staging and extensive pelvic and upper abdominal debulking cases (including bowel operations) for advanced stage cancers. Patients’ baseline characteristics, the types of surgical procedures, and the main outcome measures are listed in the Table. All pain scores were measured using a 10-point numeric pain rating scale. Chronic opioid use was defined as chronic opioid use verified by the New York State Prescription Monitoring Program Registry (I-STOP) for the 90-day preoperative period (45 patients [7.2%] in the pre-UROPP group and 61 patients [10.1%] in the post-UROPP group) (Table).

**Table.** Patient Characteristics and Outcome Measures

Characteristic	Patients, No. (%)		*P* Value
	Pre-UROPP (n = 626)	Post-UROPP (n = 605)	
Type of surgery			
Laparotomy	146 (23.3)	144 (23.8)	.97
Robotic or laparoscopic	279 (44.6)	266 (44.0)	
Ambulatory	201 (32.1)	195 (32.2)	
Age, y			
Mean (SD)	55.5 (13.9)	56.3 (14.5)	.33
Median (IQR)	56.0 (46.0-66.0)	57.0 (46.0-67.0)	
Race/ethnicity			
White	546 (87.2)	531 (87.8)	.36
African American	58 (9.3)	44 (7.3)	
Asian	8 (1.3)	9 (1.5)	
Unknown	14 (2.2)	21 (3.5)	
BMI			
Mean (SD)	33.1 (9.9)	33.0 (10.2)	.81
Median (IQR)	31.0 (26.0-38.0)	31.0 (25.0-39.0)	
Smoking status, No./total No. (%)			
Former	146/610 (23.9)	142/589 (24.1)	>.99
Yes	112/610 (18.4)	108/589 (18.3)	
No	352/610 (57.7)	339/589 (57.6)	
Prior abdominal surgical procedures, No.			
Mean (SD)	1.1 (1.3)	1.1 (1.3)	.60
Median (IQR)	1.0 (0.0-2.0)	1.0 (0.0-2.0)	
Diagnosis			
Preinvasive or benign	345 (55.1)	354 (58.5)	.23
Malignant	281 (44.9)	251 (41.5)	
Comprehensive staging performed			
Yes	114 (18.2)	98 (16.2)	.35
No	512 (81.8)	507 (83.8)	
Length of stay, d			
Mean (SD)	1.2 (1.4)	1.1 (1.5)	.79
Median (IQR)	1.0 (0.0-2.0)	1.0 (0.0-1.0)	
Chronic opioid use			
Yes	45 (7.2)	61 (10.1)	.07
No	581 (92.8)	544 (89.9)	
No. of opioid doses (intravenous and oral) during admission			
Mean (SD)	2.5 (4.5)	2.9 (5.3)	.09
Median (IQR)	1.0 (0.0-3.0)	0.0 (0.0-4.0)	
Intraoperative complications			
Yes	2 (0.3)	2 (0.3)	.97
No	624 (99.7)	603 (99.7)	
Last pain score at time of discharge^a^			
Mean (SD)	1.7 (2.4)	1.6 (2.4)	.38
Median (IQR)	0.0 (0.0-3.0)	0.0 (0.0-3.0)	
**Outcome Measures**			
Postoperative pain score at 2 wk after discharge^a^			
Mean (SD)	1.4 (2.3)	1.1 (2.2)	.06
Median (IQR)	0.0 (0.0-2.0)	0.0 (0.0-1.5)	
Patients requesting opioid refill within 30 d after surgery	104 (16.6)	100 (16.5)	.99
Postoperative complications, No./total No. (%)			
Yes	42/624 (6.7)	29/605 (4.8)	.15
No	582/624 (93.3)	576/605 (95.2)	

Abbreviations: BMI, body mass index (calculated as weight in kilograms divided by height in meters squared); IQR, interquartile range; UROPP, ultrarestrictive opioid prescription protocol.

^a^ Numerical rating of 0 to 10, where 0 is no pain and 10 is worst possible pain.

### Outcomes

Our primary outcomes were the mean postoperative pain score 2 weeks after surgery and new requests for opioid refills within 30 days after surgery, including those prescribed by an outside health care professional. All patients at the 2-week postoperative visit were asked to report their pain on a numeric scale of 0 to 10, with 0 reflecting no pain and 10 reflecting worst possible pain. All available patient records on opioid use were cross-referenced with the I-STOP registry during the perioperative period (90 days before surgery and 30 days after surgery). The I-STOP registry was partially out of reference at the time of data extraction for 119 of 626 patients (19.0%) in the pre-UROPP group but was complete for the post-UROPP group. Patients with no I-STOP data were excluded from the respective analysis.

Our secondary outcome was the number of complications within 30 days after the procedure (consistent with the American College of Surgeons National Surgical Quality Improvement Program reporting guideline^10^) (Table). Postoperative complications included any readmissions or emergency department visits related to surgery and all outpatient treatments for wound breakdown or infection. The postoperative follow-up rate was 96.3% in the pre-UROPP group (603 of 626) and 95.9% in the post-UROPP group (580 of 605) (*P* = .98).

### Statistical Analysis

The primary comparison was between the pre-UROPP and post-UROPP groups across binary, categorical, and continuous end points. All continuous variables were analyzed with a 2-sample *t* test for comparing the equality of mean values, binary variables were analyzed using the Fisher exact test for independence, and categorical end points were analyzed using the Pearson χ^2^ test for independence. All statistical analysis was performed in R, version 3.3.0 statistical language (R Foundation for Statistical Computing). All statistical tests were 2-sided, and the results were deemed statistically significant at *P* < .05. Given 626 controls and 605 cases, we had the ability to detect a 0.16-SD unit mean difference between groups at 80% power using the 2-sample *t* test. Similarly, we could detect differences in proportions between cases and controls of 0.053 to 0.081 or larger using the Fisher exact test at 80% power for control proportions ranging from 0.1 to 0.5, respectively.

## Results

During the intervention period, a total of 605 surgical patients (mean [SD] age, 56.3 [14.5] years) experienced postoperative pain that was managed using the UROPP after hospital discharge, and their outcomes were compared with those of 626 patients (mean [SD] age, 55.5 [13.9] years) from the year prior, when outpatient pain management followed no specific guidelines (controls) and thus allowed the liberal use of opioids per trainees’ and each physician’s preference. Patients’ baseline characteristics and the types of surgical procedures, which were performed by the same team of surgeons in the 2 cohorts, were not statistically different (Table). The 2 cohorts had a similarly low rate of intraoperative complications (vascular injury or ureteral injury in these cases), received the same amount of opioids while in the hospital, and had no difference in discharge pain scores. Both cohorts participated in the ERAS program (for pain management: acetaminophen, gabapentin, and cyclooxygenase-2 inhibitors preoperatively with the addition of regional anesthesia if possible or needed, and scheduled acetaminophen and nonsteroidal anti-inflammatory drugs postoperatively, if no contraindication existed).

Compliance with the protocol among the health care professionals (physicians, nurse practitioners, and physician assistants) was 605 of 627 total patients (96.5%) during the intervention period; the 22 deviations occurred mostly with ambulatory cases within the first 3 months of implementation of the protocol. The mean (SD) postoperative pain scores 2 weeks after the surgery were not statistically different between the 2 cohorts (1.1 [2.2] for the post-UROPP group vs 1.4 [2.3] for pre-UROPP group; *P* = .06), and the number of patients who requested an opioid refill within 30 days after the surgery also remained the same (104 patients [16.6%] in the pre-UROPP group vs 100 patients [16.5%] in the post-UROPP group; *P* = .99) (Table). During the 30-day postoperative period, we found no statistically significant increase in the number of postoperative complications (29 cases [4.8%] in the post-UROPP group vs 42 cases [6.7%] in the pre-UROPP group; *P* = .15) (Table).

**Figure 2.**
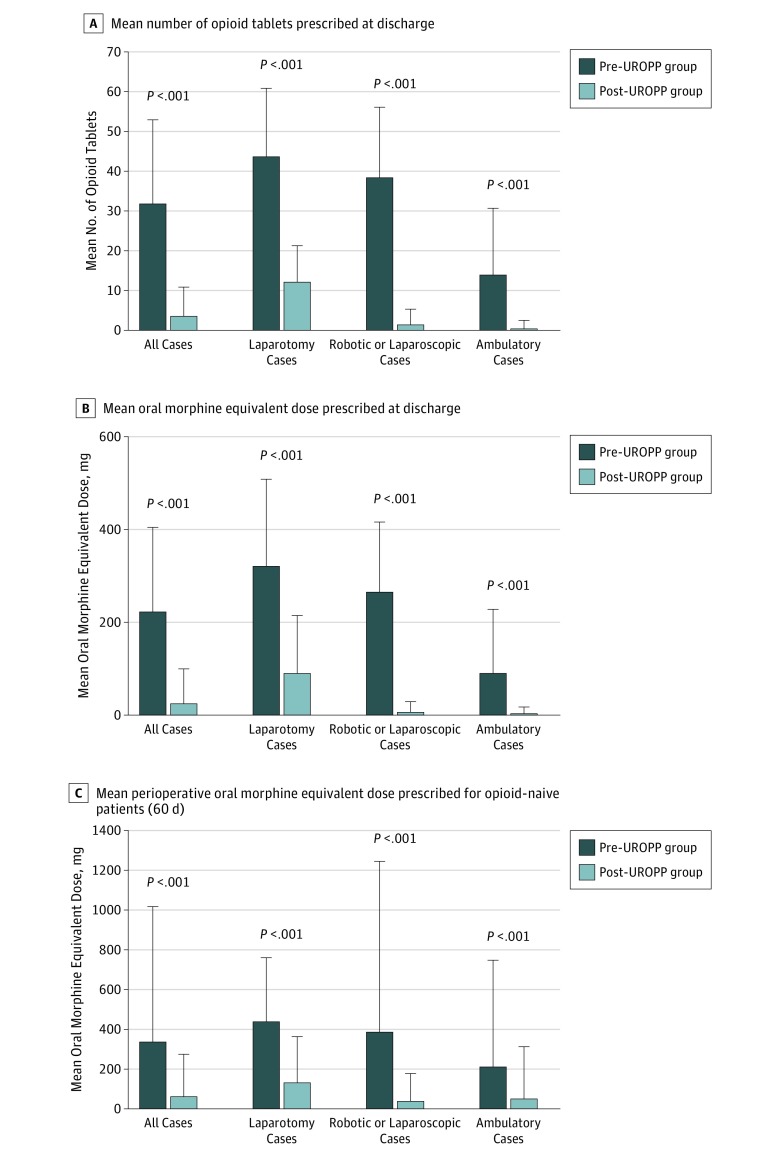
Opioids Prescribed at Hospital Discharge A, Mean (SD) number of opioid-containing tablets prescribed at hospital discharge by case types before and after implementation of the ultrarestrictive opioid prescription protocol (UROPP). B, Mean (SD) oral morphine equivalents prescribed before and after UROPP implementation for the different case types. C, Mean (SD) oral morphine equivalents prescribed during the total perioperative period (includes 30 days before surgery, discharge, and 30 days after surgery) for opioid-naive patients. Error bars represent SD.

The implementation of the UROPP was associated with a significant reduction in the mean (SD) number of opioid pills dispensed at discharge in all surgical cases in the post-UROPP group (3.5 [7.0]) compared with the pre-UROPP group (31.7 [21.0]) (*P* < .001). The mean (SD) number of opioid pills dispensed at the time of discharge decreased from the pre-UROPP period to the post-UROPP period from 43.6 (17.0) to 12.1 (8.9) in the laparotomy group (*P* < .001), from 38.4 (17.4) to 1.3 (3.7) in the MIS group (*P* < .001), and from 13.9 (16.6) to 0.2 (2.1) in the group of patients who underwent ambulatory surgery (*P* < .001) (Figure 2A). Reductions were similar in magnitude in converted oral morphine equivalents (Figure 2B). For all cases, oral morphine equivalents of 222.8 (180.2) mg vs 25.2 (71.8) mg were prescribed for pre- and post-UROPP patients, respectively. Oral morphine equivalents of 322.2 (185.1) mg vs 91.0 (122.4) mg for laparotomy cases, 266.1 (148.3) mg vs 7.0 (20.3) mg for robotic or laparoscopic cases, and 90.4 (136.5) mg vs 1.5 (14.2) mg for ambulatory cases were prescribed for pre- and post-UROPP groups, respectively. In the post-UROPP group, a hydrocodone-containing regimen was prescribed to 99 of 157 patients (63.1%) and an oxycodone-containing regimen was prescribed to 50 of 157 patients (31.8%). If a patient reported a severe allergy or refused a regimen containing hydrocodone or oxycodone, tramadol (3 of 157 [1.9%]), codeine (1 of 157 [0.6%]), or hydromorphone (4 of 157 [2.5%]) was provided. The associated reduction in total perioperative opioid use (including 30 days before surgery, discharge prescriptions, and 30 days after surgery) across all surgical cases was equally statistically significant in all subtypes of surgical procedures for opioid-naive patients (the total for all cases was reduced to a mean [SD] of 64.3 [207.2] mg after implementation of the UROPP from a mean [SD] of 339.4 [674.4] mg before implementation; *P* < .001) (Figure 2C). Breaking down to different surgical groups, oral morphine equivalents of 438.6 (318.1) mg vs 133.2 (226.4) mg for laparotomy cases, 385.8 (854.6) mg vs 39.8 (135.3) mg for robotic or laparoscopic cases, and 212.0 (532.0) mg vs 49.1 (259.5) mg for ambulatory cases were prescribed for pre- and post-UROPP groups, respectively.

**Figure 3.**
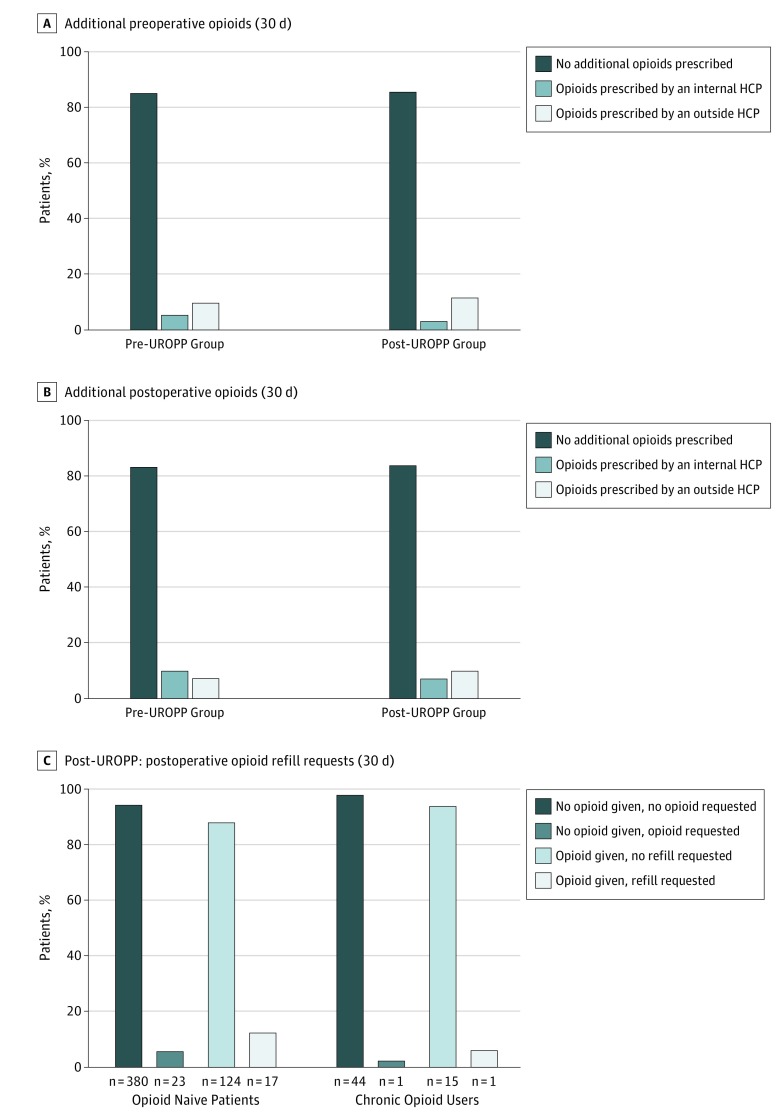
Additional Perioperative Opioid Doses and Refill Requests A, Percentage of the source of preoperative (30 days prior to surgery) opioid prescriptions in the pre-ultrarestrictive opioid prescription protocol (UROPP) and post-UROPP groups by type of health care professional (HCP). There was no statistical difference between the source of opioid prescription between the 2 cohorts of patients (*P* = .07). B, Percentage of the source of postoperative (30 days after surgery) opioid prescriptions in the pre- and post-UROPP groups by type of HCP. No statistically significant difference was seen between the 2 cohorts (*P* = .08), and there was no increase in the number postoperative opioid refills after implementation of UROPP by outside health care professionals. C, Percentage of postoperative opioid refills in post-UROPP patients based on discharge prescription status and prior opioid use.

Data from I-STOP showed that 85.0% of our surgical patients in the pre-UROPP group (431 of 507) and 85.6% of our surgical patients in the post-UROPP group (518 of 605) received no opioid prescriptions 30 days prior to surgery (Figure 3A). Preoperative opioid prescriptions were more likely to be provided by an outside health care professional rather than the surgical team in both the pre-UROPP and post-UROPP groups. After discharge in the 30-day postoperative window, 83.5% of the post-UROPP patients (505 of 605) did not request any additional opioid prescriptions (Figure 3B). This percentage of patients was similar to the prior year (83.4% [522 of 626]), when patients were discharged home with significantly more opioid tablets. The breakdown of the source of additional opioid prescriptions before implementation of UROPP was not statistically different from the breakdown of the source after implementation (Figure 3B).

When opioid-naive patients and those who were chronic opioid users were further stratified for additional opioid prescription requests in the post-UROPP group (Figure 3C), we observed that the protocol was 94.3% (380 of 403) accurate in estimating additional opioid prescription requests after the surgery by using the type of surgical intervention (MIS or ambulatory vs laparotomy) and whether patients received more than 5 opioid doses in the last 24 hours while in the hospital for triaging patients to receive an opioid prescription. Patients who were discharged home with an opioid prescription were also more likely to call and request a refill within 30 days after surgery than were patients receiving no opioids at discharge, independent of whether they were opioid naive or chronic opioid users (Figure 3C).

**Figure 4.**
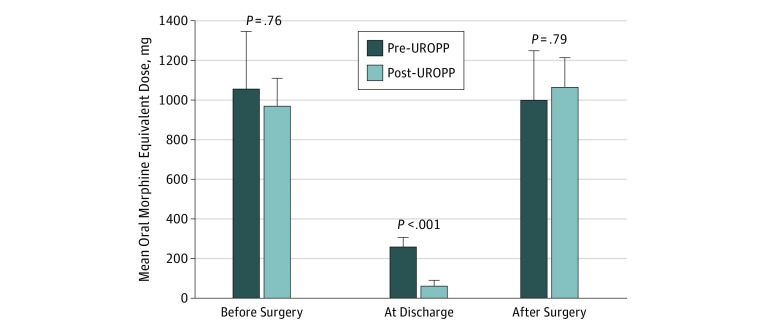
Perioperative Opioid Doses Among Chronic Opioid Users Mean (SD) total oral morphine equivalents dispensed for all cases of chronic opioid users. Using the ultrarestrictive opioid prescription protocol (UROPP) algorithm significantly decreased the dispensed oral morphine equivalents at the time of discharge in the chronic opioid user population. After surgery, chronic opioid users returned to their baseline opioid prescription refills regardless of UROPP implementation. Error bars represent SD.

Among chronic opioid users, the mean oral morphine equivalents filled 30 days before or 30 days after surgery were not statistically different in the pre-UROPP and post-UROPP groups, and patients reverted back to their baseline opioid use within 30 days after surgery. However, the oral morphine equivalents dispensed at hospital discharge were significantly reduced owing to our compliance with the protocol (Figure 4). These patients typically used higher doses of opioids while in the hospital and thus were more likely to be discharged with an opioid prescription after MIS or with a higher number of opioid pills after a laparotomy. The mean (SD) oral morphine equivalents dispensed at discharge for chronic opioid users was still statistically significantly lower than the oral morphine equivalents dispensed at discharge prior to the implementation of UROPP (64.0 [188.4] mg vs 259.5 [286.7] mg; *P* < .001).

## Discussion

These data suggest that the implementation of a UROPP in a large surgical service is feasible and safe and was associated with a significantly decreased number of opioids dispensed during the perioperative period, particularly among opioid-naive patients. After the initial intervention period, the UROPP was adopted as our standard postoperative outpatient pain management protocol, and it has been maintained without alteration for more than 1 year at our cancer center. In the last 6 months, compliance with the protocol has been 98% by our health care professionals, with no issues with sustainability. These data demonstrate that we were able to safely manage postoperative pain in patients undergoing ambulatory surgery and MIS with essentially no prescription for opioids and without adversely affecting patient recovery or pain scores or increasing the demand for additional opioids. The opioid-sparing effect was also marked and statistically significant in the laparotomy group, where most patients remained physically active and recovered well with no negative sequelae or elevated pain score after surgery.

The success of implementing this radical approach depended on the willingness of our health care team to change practice pattern and take time to educate patients preoperatively and at the time of discharge to set the right expectations for home pain management. Physician behavior profoundly influences patient care and drives opioid prescribing practices. Although there was consensus among our health care professionals regarding the implementation of the protocol, the gradual decrease in the number of protocol deviations shows the importance of data with regard to the effectiveness of opioid-sparing protocols in overcoming prior practice patterns and the fear of patient complaints.

In New York State, there are strict regulations to limit the use of opioid pain medication for acute pain after noncancer surgery^9^ to no more than a 7-day supply. Although this regulation did not affect nearly half of our surgical patients even in the pre-UROPP group owing to a diagnosis of cancer, we generally complied with the recommendation to limit the use of opioid pain medication to no more than a 7-day supply. However, our study demonstrates that even the 7-day regulation is overly generous and that stricter guidelines could even be used for many patients undergoing surgery for cancer. Most patients undergoing a wide variety of gynecologic oncology procedures had adequate postoperative pain control with just ibuprofen and acetaminophen, with the selective addition of a 3-day supply of opioids as needed. The findings also argue that previously published opioid prescribing recommendations for other surgical procedures (eg, 15 pills of opioids for laparoscopic cholecystectomy) could be more stringent, with few to no opioids given.^11,12^

We demonstrated no increase in the number of refill requests within 30 days after surgery, regardless of how many pills were dispensed at the time of discharge. These data are consistent with another publication that reported that the probability of refilling an opioid prescription after surgery did not correlate with initial prescription strength.^13^ We also found that patients who were discharged home with an opioid pain medication were more likely to request a refill despite the lack of postsurgical complications and otherwise adequate recovery, suggesting that perhaps many of these patients are using opioids for emotional coping rather than for pain management.^14^

By creating the UROPP, our goal was to develop a standardized, easy-to-use protocol for surgical services that offers the same clinical approach to opioid-naive patients and those who are chronic users. The US Food and Drug Administration states that opioid tolerance exists when a patient has used 60 mg or more of morphine equivalents per day for more than 2 weeks.^15^ However, using the standard definition of chronic pain (≥90 days), we realized that our patients with chronic pain were using opioids in such a high quantity and potency that by just lowering their discharge opioid tablet count, we could not significantly affect their overall perioperative opioid consumption (Figure 4). Even when no opioids or only a minimal amount of opioids were given to these patients, they were less likely to call for a refill (Figure 3C) because they reverted back to their preoperative baseline opioid use.

### Limitations and Strengths

In our surgical cohorts, we noted another category of patients who did not have chronic pain according to our data. However, they were using opioids before and after surgery (all prescribed by an outside health care professional). In the post-UROPP period, 20 of 266 patients (7.5%) who underwent MIS and 11 of 195 patients (5.6%) who underwent ambulatory surgery used more than 150 oral morphine equivalents for the total perioperative period, while 9 of 266 patients (3.4%) who underwent MIS and 8 of 195 patients (4.1%) who underwent ambulatory surgery consumed more than 300 mg oral morphine equivalents during the perioperative period. These patients were more likely to be discharged home with an opioid prescription per protocol because they used more opioids while in the hospital and also tended to call to request refills. These patients increased the mean perioperative opioid dose among the opioid-naive group (Figure 2C). We believe this subgroup of patients is particularly vulnerable for conversion to chronic opioid users; thus, patient education and adherence to the protocol were not only feasible but crucial to protect and achieve opioid sparing. We acknowledge this as a limitation, but, pragmatically speaking, it offers a guideline on how to manage these patients. Despite this, we found that universally applying the UROPP to all patients based on procedure type and inpatient opioid consumption (rather than classifying patients based on prior opioid use) was simple, safe, and at least 94% accurate in our cohort to determine postdischarge opioid need, and it is also more practical for larger-scale implementation. Another limitation of our study is the use of a historical control group from the year prior to implementation of the UROPP. However, our data suggest that the patient characteristics, the surgical procedures performed, and the rates of complications were similar between the 2 groups and the surgical procedures were performed by the same surgeons.

An additional limitation is that, by the nature of our practice, all the patients studied were women. Prior studies have shown that 2 major factors associated with a high rate of opioid prescriptions at discharge are female sex and moderate to severe pain scores before discharge.^16^ Studies investigating the association of sex with pain perception have also shown that women may have higher ratings of pain and lower pain tolerance, hence suggesting that extending the UROPP to male patients would be equally feasible.^17,18^ A wide range of complex pelvic and abdominal surgical procedures were performed for our patient population; thus, our results could be applied to other surgical fields. Most of our patients are elderly, are obese, and commonly present with multiple comorbidities at very difficult times in their lives, often with a diagnosis of cancer, so they may be particularly vulnerable to chronic opioid use and misuse.^19^

The strengths of our study include the large control (pre-UROPP) group with similar patient characteristics to the post-UROPP group, the ability to demonstrate the change in health care professionals’ practice patterns and to maintain sustained compliance given the lack of adverse events or increase in patients’ complaints, and the use of a state opioid monitoring program, which allows for accurate statewide tracking of dispensed opioids.

Prior publications advocating for more liberal use of opioids have argued that “sometimes less is not more”^20^^(p1189)^ and that inadequate pain management can cause unnecessary suffering and loss of function and mobility and have deleterious psychosocial and pathophysiological effects, such as postoperative ileus, impaired wound healing, pneumonia, or cardiac complications.^20,21^ We observed no increase in the number of postoperative complications in our study. From the efficacy of ERAS regarding patient recovery, one can argue that, in larger cohorts, extending opioid-sparing ERAS principles to postdischarge pain management may enhance postoperative recovery because the adverse effects of opioids can be eliminated or reduced.

## Conclusions

It is clear that chronic postoperative opioid misuse can best be addressed by preventing the initiation of opioids. As health care professionals, our primary aim is to help our patients and eliminate unnecessary treatments that may do harm. Any medication prescribed must carry benefits that outweigh the risks. Using UROPP to manage postsurgical pain after discharge did not result in any negative health consequences; thus, we advocate for adopting radical opioid-sparing approaches for managing postoperative pain nationwide. This change in practice is expected to reduce health care costs and the number of opioids circulating in our communities and will ultimately protect our patients and their family members from opioid diversion, misuse, and abuse.

## Notes

Supplement. **eTable.** Patient Characteristics and Outcome Measures **eFigure.** Opioids Prescribed on Discharge
