# Supplementary material for: Ultrarestrictive Opioid Prescription Protocol for Pain Management After Gynecologic and Abdominal Surgery
Source: JAMA Netw Open. 2018 Dec 7;1(8):e185452. doi: 10.1001/jamanetworkopen.2018.5452 (PMC6324564; doi:10.1001/jamanetworkopen.2018.5452)
Supplement: Supplement. — eTable. Patient Characteristics and Outcome Measures eFigure. Opioids Prescribed on Discharge [file jamanetwopen-1-e185452-s001.pdf]

Supplementary Online Content

Mark J, Argentieri DM, Gutierrez CA, et al. Ultrarestrictive opioid prescription protocol for pain management after gynecologic and abdominal surgery. *JAMA Netw Open.* 2018;1(8):e185452. doi:10.1001/jamanetworkopen.2018.5452

eTable. Patient Characteristics and Outcome Measures
eFigure. Opioids Prescribed on Discharge

This supplementary material has been provided by the authors to give readers additional information about their work.

© 2018 Mark J et al. *JAMA Network Open.*

eTable. Patient Characteristics and Outcome Measures

Patient Characteristics	Pre-UROPP (N = 626, 50%)	Post-UROPP (N = 627, 50%)	*P* Value
**Number of Cases**			.84
Laparotomy	146 (23.3%)	144 (23.0%)	
Robotic/Laparoscopic	279 (44.6%)	272 (43.4%)	
Ambulatory	201 (32.1%)	211 (33.6%)	
**Mean Age (SD) (years)**	55.5 (13.9)	56.2 (14.6)	.41
**Race**			.24
Caucasian	546 (87.2%)	547 (87.2%)	
African American	58 (9.3%)	46 (7.3%)	
Asian	8 (1.3%)	10 (1.6%)	
Unknown	14 (2.2%)	24 (3.8%)	
**Mean BMI (SD)**	33.1 (9.9)	33.0 (10.2)	.92
**Smoking Status**			.98
Former	146 (23.9%)	144 (23.6%)	
Yes	112 (18.4%)	114 (18.7%)	
No	352 (57.7%)	352 (57.7%)	
**Mean Prior Abdominal Surgeries (SD)**	1.1 (1.3)	1.2 (1.3)	.53
**Diagnosis**			.13
Pre-invasive/Benign	345 (55.1%)	372 (59.3%)	
Malignant	281 (44.9%)	255 (40.7%)	
**Comprehensive Staging Performed**			.25
Yes	114 (18.2%)	99 (15.8%)	
No	512 (81.8%)	528 (84.2%)	
**Mean Length of Stay (SD) (days)**	1.2 (1.4)	1.1 (1.5)	.49
**Chronic Opioid Use**			.05
Yes	45 (7.2%)	65 (10.4%)	
No	581 (92.8%)	562 (89.6%)	
**Mean Number of Opioid Doses (Intravenous and Oral) During Admission (SD)**	2.5 (4.5)	2.9 (5.2)	.16
**Intraoperative Complications**			1.00
Yes	2 (0.3%)	2 (0.3%)	
No	624 (99.7%)	625 (99.7%)	
**Mean last pain score at time of discharge (SD)***	1.7 (2.4)	1.6 (2.4)	.41
**Outcome Measures**			
**Mean postoperative pain score 2 weeks after discharge (SD)***	1.4 (2.3)	1.1 (2.2)	.11
**Number of patients requesting opioid refill within 30 days after surgery (%)**	104 (16.6)	105 (16.7)	.99
**Postoperative Complications**			.22

© 2018 Mark J et al. *JAMA Network Open.*

Yes	42 (6.7%)	32 (5.1%)	
No	582 (93.3%)	595 (94.9%)	

* Pain Score: 0-10 numbered rating, where 0 is no pain and 10 is worst possible pain.

Previously excluded 22 patients for protocol violations have been included in this table. Patients' baseline characteristics and outcome measures between Pre-UROPP and Post-UROPP cohorts were not statistically different with exception of more chronic opioid users in the Post-UROPP group (*P*=.05).

© 2018 Mark J et al. *JAMA Network Open.*

eFigure. Opioids Prescribed on Discharge

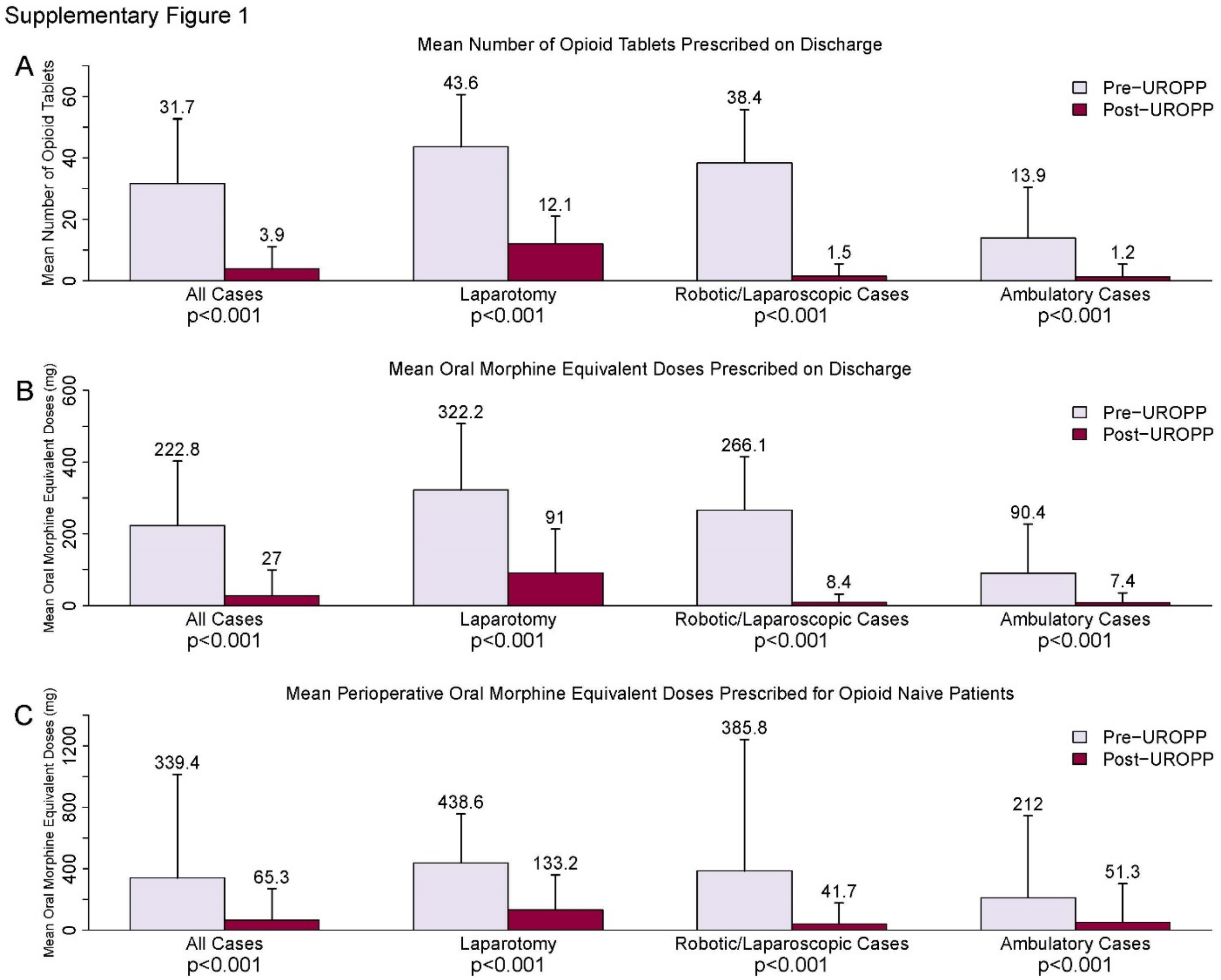

Previously excluded 22 patients for protocol violations have been included in this table **A)** The mean number of opioid containing tablets prescribed on hospital discharge by case types before and after UROPP implementation. The implementation of the UROPP led to a statistically significant reduction in prescribed opioids in all case types (P<.001). **B)** Mean oral morphine equivalents (OME) prescribed before and after UROPP implementation in the different case types. **C)** Mean OME prescribed in the total perioperative period (includes 30 days before surgery, discharge, and 30 days after surgery). All pairwise comparisons showed significant (P<.001) reduction in dispensed post-UROPP OME. Error bars represent the standard deviation (SD).

© 2018 Mark J et al. *JAMA Network Open.*
